# Effects of Gender and Personality on First Impression

**DOI:** 10.1371/journal.pone.0135529

**Published:** 2015-09-02

**Authors:** Katia Mattarozzi, Alexander Todorov, Michele Marzocchi, Alba Vicari, Paolo Maria Russo

**Affiliations:** 1 Department of Experimental, Diagnostic and Specialty Medicine, University of Bologna, Bologna, Italy; 2 Department of Psychology, Princeton University, Princeton, New Jersey, United States of America; 3 Department of Psychology, University of Bologna, Bologna, Italy; Ecole Normale Supérieure, FRANCE

## Abstract

The present study explores whether and to what extent individual differences (i.e., gender and personality traits of perceiver) predict inferences of trustworthiness from emotionally neutral unfamiliar faces and the related confidence in judgment. Four hundred and ten undergraduate students participated in the study. Personality was assessed using the Big Five model (i.e., Extraversion, Neuroticism, Conscientiousness, Agreeableness and Openness to experience) and measures of trait anxiety and aggression. The results suggest that trustworthiness judgments are affected by the gender of the perceiver, although this effect depends on the valence of the face. Women tend to judge trustworthy-looking faces as significantly more trustworthy than men do, and this is particularly pronounced for judgments of female faces. There were no gender differences for judgments of untrustworthy-looking or neutral faces. Gender also seems to affect the confidence in judgment. Specifically, women were generally less confident than men in judging trustworthiness of unfamiliar faces. Personality also affected judgment. Both low agreeable individuals and high trait aggressive individuals tend to perceive unfamiliar faces as less trustworthy. The present findings suggest that both gender and personality traits are relevant for understanding how people evaluate the trustworthiness of others. Whom we decide to trust is a function not only of their facial features but also of gender and individual differences in personality traits.

## Introduction

The decision of whom to trust or not is a fundamental aspect of social interaction. Such decisions are often based on as little information as first impressions from facial appearance [1–5]. A series of recent studies shows that even emotionally neutral faces are automatically evaluated on valence, which is best approximated by judgments of trustworthiness [6–9]. These evaluations are made rapidly and effortlessly [10] and, although not necessarily accurate, they affect motivation, feelings and decision making [1, 2, 11].

Although evaluations from faces are fairly consistent across perceivers, the consistency is far from perfect [12, 13]. Individual differences, such as gender or personality traits, may contribute to differences in face evaluation. There is substantial evidence indicating a female advantage in processing face-specific information. In particular, it seems that women have superior ability in identity discrimination [14, 15] and are faster and more accurate than men at recognizing emotional expressions [16, 17]. This female advantage seems to emerge especially when the emotional expression is subtle [18–20]. Moreover, women are more defensively reactive (i.e., greater facial EMG activity, greater bradycardia, larger electrodermal responses, increased startle reflex) to aversive stimuli and rate emotional pictures more extremely on hedonic valence than men [21, 22]. Several studies suggest that valence evaluation of emotionally neutral faces involves an overgeneralization of adaptive mechanisms underlying the processing of emotional faces [6, 11, 23, 24]. Specifically, emotionally neutral faces that resemble emotional expressions (e.g., anger) are judged in accordance with these expressions (e.g., aggressive). Therefore, we might expect that gender also influences judgments of unfamiliar, emotionally neutral faces.

It is well known that affective information processing is affected by personality traits such as neuroticism, extraversion, negative affect, anxiety, and aggression [25, 26]. Individuals characterized by a high degree of neuroticism (compared to emotionally stable individuals) have a greater defensive activation to aversive stimuli [27, 28]. Extraverts (compared to introverts) show heightened emotional reactivity to positive (but not negative) signals of reward [27]. With respect to trustworthiness judgments from facial appearance, recent studies [29, 30] showed that higher levels of trait anxiety and aggression were associated with judgments of faces as less trustworthy.

The present study explores whether and to what extent individual differences (i.e., gender and personality traits of perceiver) predict inferences of trustworthiness from emotionally neutral faces and the related confidence in judgment. Personality was assessed using the Big Five model (for an historical overview, see De Raad, 2003 [31]). Specifically, we examined the potential contribution of the Big Five factors (i.e., Extraversion, Neuroticism, Conscientiousness, Agreeableness and Openness to experience) to trustworthiness judgments of unfamiliar faces. In addition, as in recent studies [29, 30], we investigated the role of trait anxiety and aggression. Since we hypothesized that a more intuitive, pre-conscious, affective, holistic approach style of information processing would lead to a greater confidence in trustworthiness judgments, the two cognitive styles (Need of Cognition and Faith of Intuition) postulated by the Cognitive-Experiential Self-Theory [32] were also included as personality measures.

In line with the above-mentioned studies on gender and personality differences in information processing of affective stimuli, we expected that: a) women would make more extreme trustworthiness judgments (in both positive and negative directions) than men; b) personality dispositions would influence trustworthiness judgments so that trait anxiety and aggression would be negatively associated with these judgments whereas agreeableness would be positively associated; c) more intuitive participants, with an affective and holistic approach to information processing, would be more confident in their judgments than those with a more rational and analytical approach.

## Material and Methods

### Subjects

Four hundred and ten undergraduate students (244 women) from the University of Bologna, participated voluntarily (mean age ± SD: 22,82 ± 5,45). All were native Italian speakers with normal or corrected-to-normal vision. Participants gave their oral consent to participate in the study.

### Stimuli

Sixty-three color-pictures of real, emotionally neutral faces were selected from the Karolinska Faces Database [33]. These faces were rated on a number of personality traits provided by the Social Perception Lab database [6]. Sixty faces were used as target stimuli, twenty of them were classified as trustworthy (standardized z scores mean ± SD: 0,67 ± 0,22; 7 males and 13 females), 20 were classified as neutral (standardized z scores mean ± SD: -0,02 ± 0,11; 10 males and 10 females), and 20 were perceived as untrustworthy (standardized z scores mean ± SD: -0,68 ± 0,41; 14 males and 6 females). Three faces served for practice trials (one trustworthy-looking, one neutral trustworthy and one untrustworthy-looking).

### Questionnaires

Personality was assessed with the following questionnaires:

- Big Five Questionnaire (BFQ) [34]. The BFQ is the most widely used measures of the Big Five personality dimensions in the Italian context and is aligned with other Big Five measures [35]. The short form of the BFQ, used in the present study, consists of 60 items, with 12 items for each dimension: (1) Energy/Extraversion, comprising activity, assertiveness, and self-confidence; (2) Agreeableness, including sympathy, kindness, and sensitiveness toward others; (3) Conscientiousness, pertaining to self-regulation in both proactive and inhibitory modes; (4) Emotional Stability, pertaining to the ability of copying/controlling anxiety and emotions; (5) Openness, pertaining to the propensity for having wide interests and for being imaginative and insightful. Respondents indicate the extent to which each item describes themselves on a 5-point scale ranging from complete disagreement (1 = very false for me) to complete agreement (5 = very true for me). Cronbach’s alpha coefficients of the five scales, obtained in the present study, range from 0.60 to 0.75.
- State-Trait Anxiety Inventory-Y (STAI-Y) [36]. The STAI-Y is the most widely used measure of state and trait anxiety; it assesses state anxiety with 20 statements that participants evaluate with respect to how they feel ‘‘right now, at this very moment”; whereas trait anxiety is assessed with 20 statements that participants evaluate with reference to how they ‘‘generally” feel. Responses are made on a 4-point Likert scale, ranging from "Not At All" to "Very Much So". Total scores for State and Trait Anxiety range from 20 to 80, with higher scores denoting higher levels of anxiety. Cronbach’s alpha coefficients were 0.93 and 0.91 for the State and Trait scales, respectively.
- Aggression Questionnaire (AQ) [37, 38]. The AQ questionnaire consists of 29 items evaluating 4 dimensions: Physical Aggression (9 items), Verbal Aggression (5 items), Anger (7 items) and Hostility (8 items). Items were answered on a 5-point Likert scale where 1 represented “Entirely false for me” and 5 represented “Entirely true for me”. The questionnaire yields four subscale score, which can be summed for a total aggression score. Cronbach’s alpha coefficients range from 0.58 to 0.83.
- Rational-Experiential Inventory (REI) [39]. Based on the Cognitive-Experiential Self-Theory [40], the REI assesses two cognitive styles: a rational style, measured by an adapted Need for Cognition (NFC) scale [41], that emphasizes a conscious, analytical approach (e.g. "I would prefer complex to simple problems") and b) an experiential style, measured by the Faith in Intuition (FI) scale, that refers to a pre-conscious, affective, holistic approach ("My initial impressions of people are almost always right"). In the present study, the 10 items version of the scale was used [32] with 5 items for each dimension. Cronbach’s alpha coefficient for the NFC and FI scales was 0.78 and 0.71, respectively.

### Procedure

The study was conducted in groups that ranged in size from 15 to 60 participants. The main investigators explained the study to the potential participants verbally, providing all pertinent information (purpose, procedures, risks, benefits, alternatives to participation, etc.), and we allowed the potential participants ample opportunity to ask questions. Following this verbal explanation, potential participants were provided with a written summary of the study and were given sufficient time to consider whether or not to participate in the study.

After allowing the potential participants time to read the study information sheet, the main investigators answered any additional questions and obtained verbal agreement to participate in the research. Students, who consented to participate, received a numeric code (e.g. 001; 002) and an envelope containing paper questionnaires and task answer sheets. The study and consent procedure was approved by the IRB of the University of Bologna. We used the oral consent procedure because: (i) the research involves no more than minimal risk (i.e., breach of confidentiality); and (ii) the only record linking the subject and the research would be the consent document and the principal risk would be potential harm resulting from breach of confidentiality.

The experiment consisted of two parts, the order of which was counterbalanced across the groups of participants. One part consisted of the administration of the questionnaires and the other consisted of the trustworthiness and confidence judgment task. Faces were presented for 3 seconds each. The trial presentation order was randomized with the restriction that the same face valence or gender did not occur on more than two consecutive trials. The presentation order of the sets was also counterbalanced across the groups of participants. Participants were asked to judge the faces on trustworthiness on a 9-point scale, ranging from 1 (not at all trustworthy) to 9 (extremely trustworthy). Immediately after the trustworthiness rating of the face, participants were asked to report their confidence in the judgment on a scale, ranging from 1 to 9.

The task started with a few practice trials to make the participant familiar with the task. Each trial was preceded by a fixation cross for 8 seconds. The size of the slide image projected centrally on a projection screen was approximately 1.2 m x 1.8 m.

## Results

Preliminary one-way ANOVAs were conducted on each personality questionnaire subscale score, with *P* values adjusted using the Bonferroni method (alpha values was set at .0036, i.e. 0.05/14 comparisons). The results showed that men and women significantly differed on, Emotional Stability [F_(1,408)_ = 5.09; p < .001, η_p_^2^ = .03], and Physical Aggression [F_(1,408)_ = 23.73; p < .001, η_p_^2^ = .05]. Table 1 lists the mean scores (and standard deviations) obtained by the two groups on each personality questionnaire.

**Table 1:** Mean (± SD) age and score obtained by Men and Women in each personality questionnaires.

	Men *(n = 166)*	Women *(n = 244)*	*p* *
**AGE**	22.78 ± 5.09	22.86 ± 5.68	n.s.
**BFQ**			
Energy	3.23 ± 0.50	3.12 ± 0.47	p = .033
Agreeableness	3.31 ± 0.44	3.44 ± 0.47	p = .005
Conscientiousness	3.41 ± 0.53	3.50 ± 0.55	n.s.
Emotional Stability	3.17 ± 0.61	2.94 ± 0.60	**p < .001**
Openness	3.40 ± 0.58	3.28 ± 0.58	n.s.
**AQ**			
Physical Aggression	19.34 ± 7.08	16.11 ± 6.22	**p < .001**
Verbal Aggression	14.82 ± 3.58	14.29 ± 3.37	n.s.
Anger	16.5 ± 5.50	17.15 ± 5.50	n.s.
Hostility	21.42 ± 5.85	21.9 ± 6.32	n.s.
**STAI-Y**			
State Anxiety	38.47 ± 10.8	39.45 ± 10.55	n.s.
Trait Anxiety	43.47 ± 9.72	45.96 ± 10.76	p = .017
**REI**			
Need for Cognition	18.88 ± 3.73	19.52 ± 3.19	n.s.
Faith in Intuition	17.30 ± 3.33	17.55 ± 3.25	n.s.

* *p* = .*0036 was accepted as statistically significant adjusting with Bonferroni method*.

### Trustworthiness Judgments

To test whether the type of face (i.e., the intrinsic valence and the gender of the face) and the gender of the perceiver affected the trustworthiness judgment of the face, we performed a mixed-model 3x2x2 ANOVA, with Facial Appearance / intrinsic valence (as a within-subject factor, 3 Levels: Trustworthy vs. Neutral vs. Untrustworthy), Face Gender (as a within-subject factor, 2 Levels) and Gender of the Perceiver (as a between-subject factor, 2 Levels), and trustworthiness judgments as a dependent variable.

As expected, a significant main effect of Facial Appearance [F_(2,816)_ = 1267.18; p < .001, η_p_^2^ = .77] confirmed that the selected stimuli significantly differed on perceived trustworthiness. Specifically, untrustworthy faces (4.25±0.94) were judged more negatively than both trustworthy (5.96 ±0.88; p < .001) and neutral (5.19±0.90; p < .001) faces. Trustworthy faces were judged more positively than neutral faces (p < .001). The effect of Facial Appearance was modulated by the Gender of the Face [F_(1,408)_ = 10.46, p = .001, η_p_^2^ = .02]. More precisely, trustworthy female faces were judged as more positive (6.09±0.94) compared to trustworthy male faces (5.72±0.98), while untrustworthy female faces (4.10±1.10) were judged as more negative compared to untrustworthy male faces (4.31±0.98).

**Fig 1:**
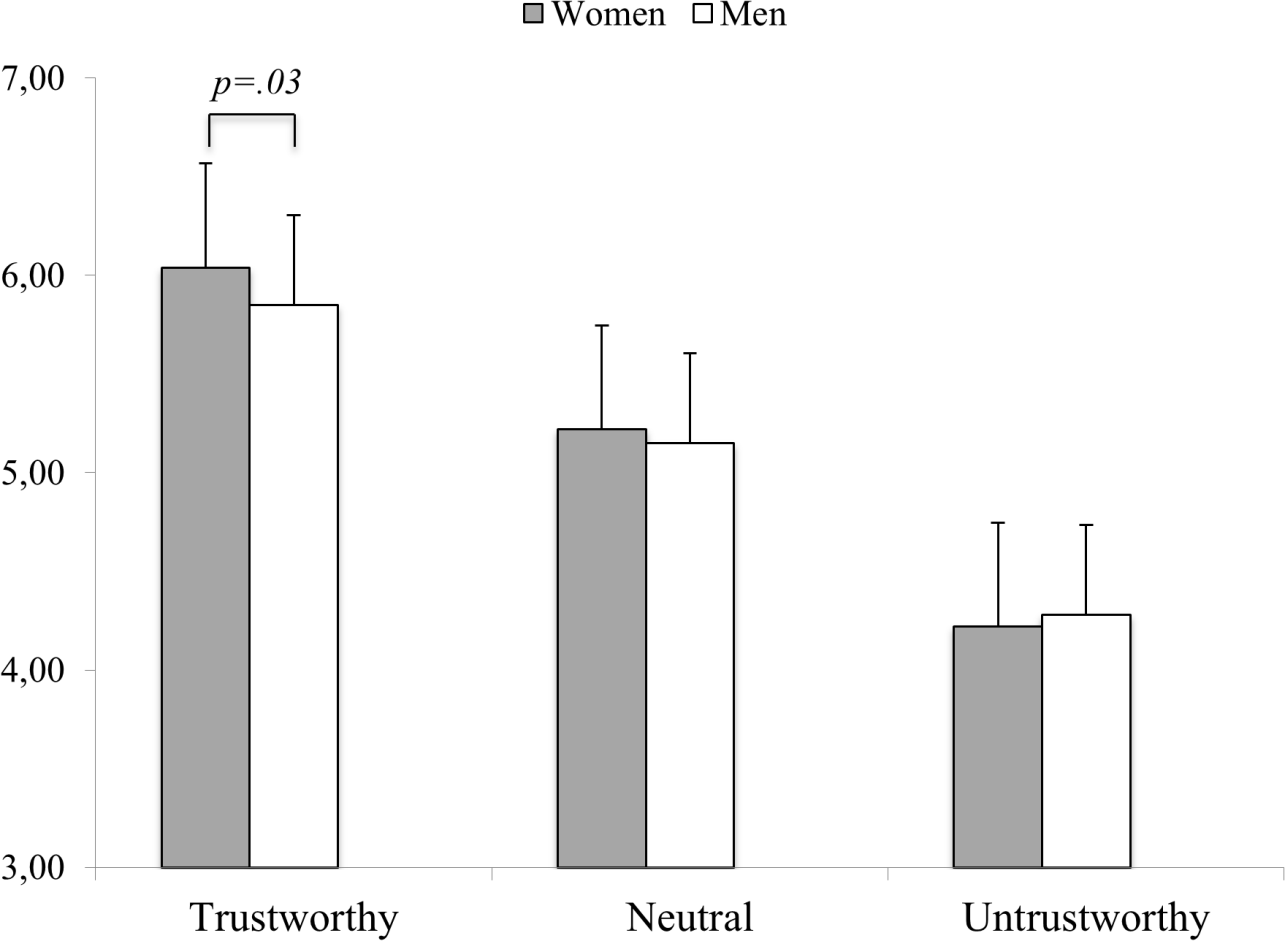
Face Trustworthiness Judgments as a function of Facial Appearance and the Gender of the Perceiver. Error bars represent standard error of the mean.

Importantly, men and women differed in their trustworthiness judgments across the three classes of Facial Appearance [Facial Appearance x Gender of the Perceiver: F_(2,816)_ = 4.74, p = .014, η_p_^2^ = .01]. Specifically, compared to men, women gave more positive judgments to trustworthy faces (6.04±0.88 vs. 5.85±0.86). For neutral or untrustworthy faces, no significant gender differences emerged (see Fig 1).

**Fig 2:**
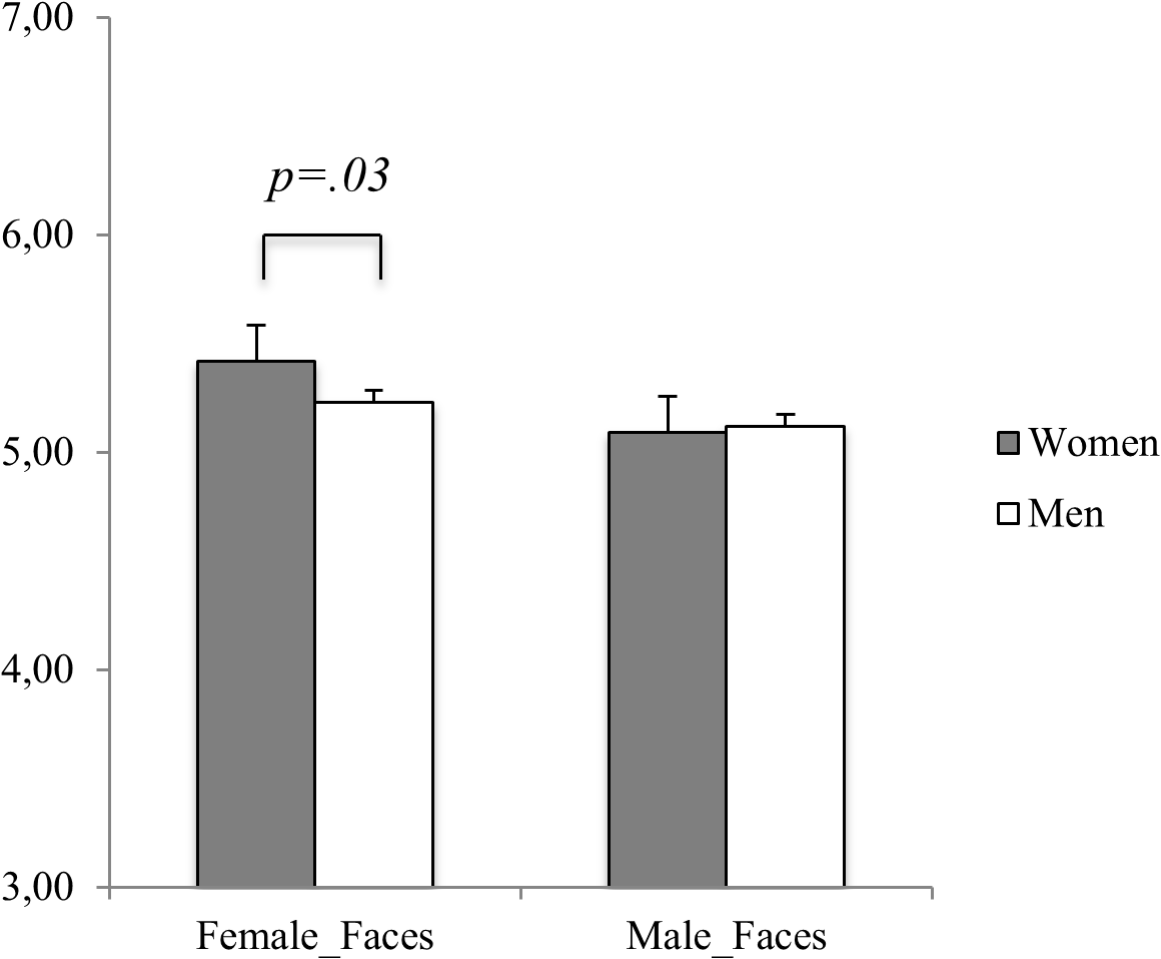
Face Trustworthiness Judgments as a function of the Gender of the Face and the Gender of the Participant. Error bars represent standard error of the mean.

Furthermore, women, regardless of Facial Appearance, rated female faces (5.42±0.88) as more trustworthy than did men (5.09±0.88), whereas men and women did not differ in their trustworthiness ratings of male faces [Face Gender x Gender of the Perceiver: F_(1,408)_ = 10.46, p = .001, η_p_^2^ = .02]. This interaction effect is depicted in Fig 2.

It is important to note that when personality traits, which differed between men and women (i.e., Emotional Stability and Physical Aggression), were included in an ANCOVA design as covariates, the above results were confirmed [Facial Appearance F_(2,658)_ = 41.32, p < .001, η_p_^2^ = .09; Facial Appearance x Gender of the Perceiver F_(2,658)_ = 4.00, p = .026, η_p_^2^ = .01; Face Gender x Gender of the Perceiver F_(1,406)_ = 9.20, p = .003, η_p_^2^ = .02], with the only exception of the interaction between Facial Appearance and Face Gender [F_(2,809)_ = 2.18, p = .11, η_p_^2^ = .01], which did not reach significance in this analysis. Tests of collinearity using tolerance and the variance inflation factor (VIF) showed no signs of collinearity, with a tolerance value close to one and low VIF values.

In order to assess the relationship between trustworthiness judgments and personality traits, Pearson’s correlation coefficients were computed both for total sample and for men and women separately. Table 2 summarizes the results.

**Table 2:** Pearson’s correlation coefficients among personality traits and both trustworthiness and confidence judgments. Data are reported for the total sample (upper line), and separately, for Men and Women (line below). Values in bold indicate significant correlations at p < .05.

	Trustworthiness Judgment	Confidence Judgment
**BFQ**		
Energy/Extraversion	-.06 (♀:-.10; ♂: .02)	**.12** (♀: **.13**; ♂: .07)
Agreeableness	**.15** (♀: **.14**; ♂: **.16**)	-.07 (♀:-.04; ♂:-.07)
Conscientiousness	-.05 (♀:-.04; ♂: .04)	-.03 (♀:-.03; ♂: .01)
Emotional Stability	.07 (♀: .06; ♂: .11)	.04 (♀: .04; ♂:-.03)
Openness	.03 (♀: .07; ♂:-.02)	-.02 (♀:-.03; ♂:-.03)
**AQ**		
Physical Aggression	-.07 (♀:-.12; ♂: .02)	**.14** (**♀: .14**; ♂: .05)
Verbal Aggression	**-.12** (**♀:-.20**; ♂:-.01)	.05 (♀: .02; ♂:-.04)
Anger	-.07 (**♀: -14**; ♂: .03)	.05 (♀: .08; ♂: .02)
Hostility	**-.11** (**♀:-.14**; ♂:-.06)	.02 (♀: .05; ♂:-.03)
AQ Total Score	**-.12** (**♀:-.18**; ♂:-.01)	.09 (**♀: .**11; ♂:-.03)
**STAI-Y**		
State Anxiety	-.07 (♀:-.07; ♂:-.08)	**-.14** (**♀:-.20**; ♂:-.02)
Trait Anxiety	-.06 (♀:-.06; ♂:-.07)	**-.13** (**♀:-.15**; ♂:-.06)
**REI**		
Need for Cognition	-.04 (♀:-.01; ♂:-.11)	-.01(♀:-.05; ♂: .08)
Faith in Intuition	-.06 (♀:-.09; ♂:-.03)	**.22** (**♀: .28**; ♂: .14)

As expected, Agreeableness was positively correlated with trustworthiness judgments, confirming that, as postulated in the Big Five model, people who score high on this dimension tend to perceive an unfamiliar face as more trustworthy. Furthermore, low but statistically significant correlations were evidenced between trustworthiness judgments and aggression traits; in particular, between trustworthiness judgments and Aggression Total Score, Hostility and Verbal Aggression. It is noteworthy that correlations between perceived trustworthiness and aggression scores vary as a function of gender (i.e., the correlation coefficients are statistically significant only for women). In order to investigate whether Gender of the perceiver interacts with Personality Traits in predicting Trustworthiness judgments, we conducted a separate regression analysis for each personality trait, using Trustworthiness rating as a criterion variable and Gender of the Perceiver, Personality Trait and Gender of the Perceiver x Personality Trait interaction term as predictors. These analyses revealed a statistically significant interaction of Gender of the Perceiver x Verbal Aggression (Standardized Beta = -.43; t = -2.01; p = .045) and marginally significant interactions for Gender of the Perceiver x AQ Total score (Standardized Beta = -.39; t = -1.74; p = .082), and Gender of the Perceiver x Hostility (Standardized Beta = -.30; t = -1.71; p = .087).

### Confidence Judgments

The same 3x2x2 ANOVA model used to analyze the trustworthiness judgments was used for the analysis of the confidence judgments. The results showed a significant main effect of Facial Appearance [F_(2,816)_ = 35.87, p < .001, η_p_^2^ = .08]. Trustworthy faces were judged with high confidence (6.38±1.18) compared to both neutral (6.16±1.22; p < .001) and untrustworthy faces (6.10±1.32; p < .001) There was no significant difference between confidence judgments of untrustworthy and neutral faces. Furthermore, confidence judgments varied across the three classes of faces depending on the gender of the face [Facial Appearance x Face Gender, F_(2,816)_ = 11.755, p < .001, η_p_^2^ = .03]. Specifically, female trustworthy faces were judged with more confidence than male trustworthy faces (6.44±1.18 vs. 6.25±1.26, respectively; p < .001), while no significant gender differences were observed for neutral and untrustworthy faces.

As revealed by the significant main effect of Gender of the Perceiver [F_(1,408)_ = 10.20, p = .002, η_p_^2^ = .02], women were less confident than men (6.07±1.24 vs. 6.44±1.10, respectively). Moreover, the difference between men and women was particularly pronounced for judgments of untrustworthy faces [Facial Appearance x Gender of the Perceiver: F_(2,816)_ = 3.688, p = .034, η_p_^2^ = .01]. Post hoc Bonferroni tests indicated that, unlike men, women were slightly less confident in judging untrustworthy faces than neutral ones (6.00±0.8 vs. 5.91±0.9, respectively; p < .05), while this difference was not significant for men (6.39±0.9 vs. 6.37±0.9, for neutral and untrustworthy faces, respectively; n.s.). In any case, both men and women, as mentioned above, tend to be less confident in judging untrustworthy faces compared to trustworthy faces.

Moreover, participants were more confident in judging female faces compared to male faces [Face Gender: F_(1,408)_ = 12.691, p < .001, η_p_^2^ = .03; 6.28±1.18 vs. 6.17±1.23, respectively]. Interestingly, a significant interaction between Face Gender and Gender of the Perceiver emerged from the analyses [F_(1,408)_ = 6.767, p = .010, η_p_^2^ = .02]. Women were more confident in judging trustworthiness of female faces compared to male faces (6.15±1.22 vs. 5.99 ± 1.28; p < .001), while the confidence judgments given by men did not differ as a function of the gender of the face (6.44±.09 vs. 6.42±0.89; n.s.).

**Fig 3:**
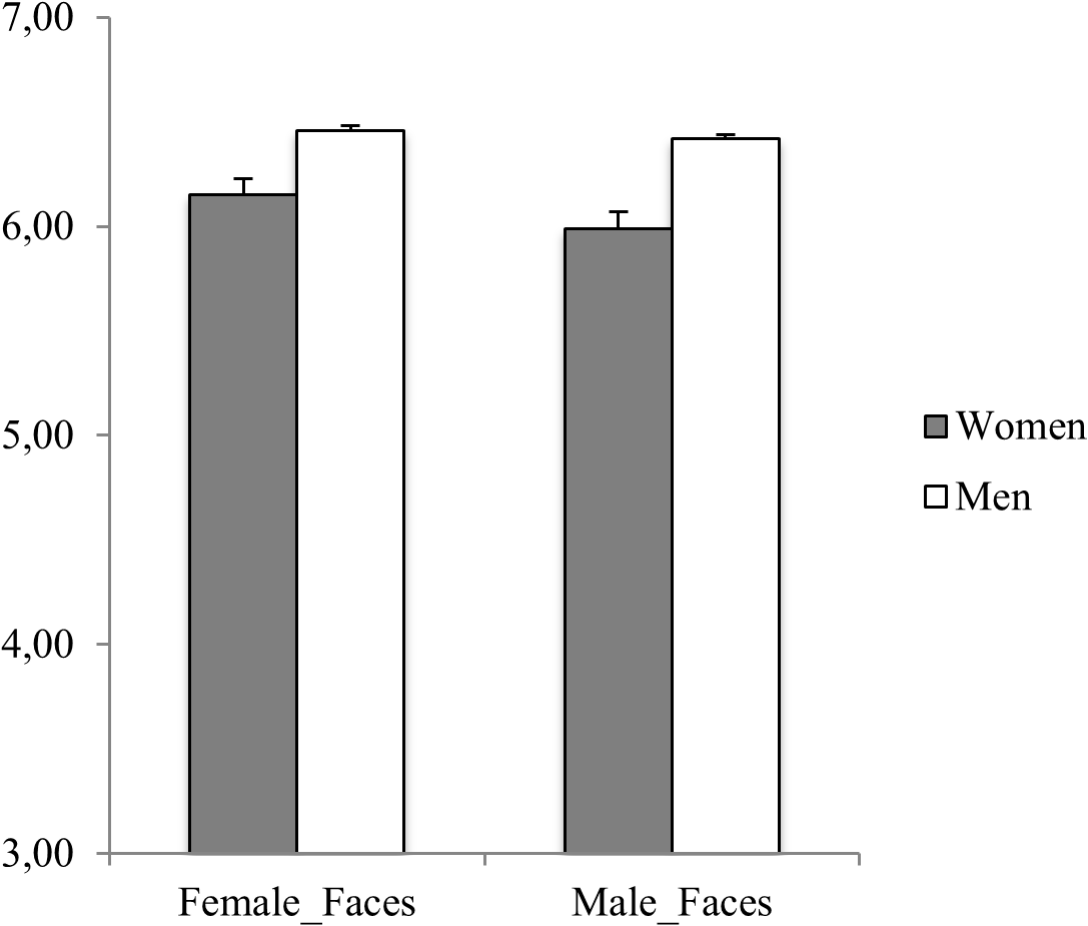
Confidence Judgments as a function of the Gender of the Face and the Gender of the Participant. Error bars represent standard error of the mean.

It is important to note that when Emotional Stability and Physical Aggression were included in an ANCOVA design as covariates, the effects of Facial Appearance (both as a main effect and in interaction with the others factors) were no longer significant. However, even controlling for differences in personality dispositions, the confidence in judgment changed significantly according to the Gender of the Perceiver and the Gender of the Face as described above [Face Gender x Gender of the Perceiver: F_(1,406)_ = 6.26, p = .013, η_p_^2^ = .02]. Fig 3 summarizes the main results for confidence judgments.

In order to assess the relationship between confidence judgments and personality traits, we conducted Pearson’s correlation analyses. These analyses showed low, but significant, correlations between confidence judgments and State and Trait Anxiety, indicating that anxious participants report lower confidence in their judgments. Furthermore, a significant positive correlation was evidenced between Faith in Intuition and confidence judgments. Precisely, more intuitive individuals were more confident in their judgments. Low size positive correlations also emerged between confidence judgments and both Energy/Extraversion and Aggression related dimensions (see Table 2).

## Discussion

In the present study, we explored the relationship between individual differences (i.e., gender and personality traits) and trustworthiness judgments of unfamiliar and emotionally neutral faces. The results suggest that these judgments are affected by the gender of the perceiver, although this effect depends on the valence of the face. Women tend to judge trustworthy-looking faces as significantly more trustworthy than men do. There were no gender differences for judgments of untrustworthy-looking or neutral faces. Moreover, unlike men, women’ trustworthiness judgments are also affected by the gender of the face. Specifically, women judge faces of other women as slightly more trustworthy compared to male faces.

The majority of studies addressing gender differences focused mainly on emotion recognition from facial expression, and very few studies have examined the potential contributions of individual differences to trustworthiness judgment of an unfamiliar, emotionally neutral faces. A previous study showed that demographic variables, including gender, do not seem to influence judgments of trustworthiness and approachability [30]. However, there is some electrophysiological evidence suggesting that there may be differences in the way facial trustworthiness is processed by women and men [42]. Moreover, a recent study [43] showed different adaptation effects on perception of face trustworthiness (i.e. visual aftereffects) for women and men. Consistent with these studies, our findings suggest that gender is a factor that should be considered in studies on first impressions. Critically, however, the obtained effects are rather small and, consequently, caution is required in interpreting gender effects.

The gender differences in judgments of trustworthy faces observed here could be interpreted as an effect of subtle variations in the features of the face that contribute to perceptions of trustworthiness. Based on behavioral studies and computational modeling, Todorov and colleagues argued that as faces move from the negative to the positive extremes of the trustworthiness dimensions, their expressions change from expressing anger to expressing happiness, and, at the same time, they change from feminine and baby-faced to masculine and mature-faced [6]. Specifically, trustworthy faces are characterized by configurations of physical features similar to babies’ faces such as large, bulbous forehead, large eyes, a small mouth, chubby cheeks, and close-set facial features. Studies examining gender differences in processing of infant faces found that women are better able to discriminate small changes in cuteness of infant faces than men [44, 45]. Based on these studies, we may hypothesize that women tend to judge unfamiliar trustworthy faces as more trustworthy because this type of face shares physical features with baby faces. These findings seem to suggest that trustworthy faces, like baby faces, are able to differentially activate the appetitive system (and, at the same time, inhibit the aversive system) in men and women.

We did not observe gender differences for judgments of untrustworthy faces. Although gender differences are observed for highly arousing, negative-affect emotional pictures, such differences tend to disappear for low arousal images [21].

Concerning the effect of personality differences on first impression, the results of the present study indicate that Agreeableness and Aggression significantly affect judgments of trustworthiness. With respect to Agreeableness, to the best of our knowledge, no study has previously investigated the relation between Big Five dimensions and perceived trustworthiness. The observed positive association between Agreeableness and perceived trustworthiness is consistent with the definition of this superordinate trait as an aggregation of lower-level traits such as trust, straightforwardness, altruism, compliance, modesty, and tender-mindedness. Likewise, in the Big Five model, low agreeableness is often characterized by skepticism about other people's motives resulting in suspicion, unfriendliness, and, as confirmed by the present study, the tendency to perceive unknown people as untrustworthy.

The present study also suggests that more aggressive people tend to perceive unfamiliar faces as less trustworthy. In line with existing evidence [46–48] and our hypothesis, this finding indicates that emotionally-neutral or ambiguous stimuli are differently evaluated by high and the low trait aggressive individuals. Furthermore, the present results confirm those obtained by Knyazev and coworkers (2008) [29] in which individuals high on aggression perceived neutral facial expressions as less friendly. Interestingly, in the study of Knyazev et al. (2008) [29], hostility-related differences were more pronounced (in both behavioral and cortical oscillatory responses) in women than in men. This result is consistent with the present findings and indicates that individual differences in aggression seem to have an impact on trustworthiness judgments in women but not in men.

With respect to the anxiety trait, these findings seem to be inconsistent with the results from a previous study [30], which suggested that trait anxiety is a significant predictor of trustworthiness judgments (i.e. individuals with higher levels of trait anxiety perceive affectively neutral faces as less trustworthy than those with lower levels of trait anxiety). In the present study, we did not replicate this finding, although our sample was larger. Methodological differences between the two studies may explain, at least in part, the differences in the results obtained. A potentially important difference concerns the task used. In the study by Willis and coworkers [30], trustworthiness was measured by presenting a face in a specific situation (i.e. participants were told to imagine being on a crowded street while on holiday and were asked to indicate whether they would trust a stranger that offers to take a photograph of them in front of a monument with their camera). Thus, it is likely that referring to such specific situations was more effective to elicit the anxiety related differences in trustworthiness judgments than the standard procedure used in the present study, in which participants were asked to evaluate trustworthiness of a face presented without contextual information.

Our results further suggest that evaluation of trustworthiness from facial appearance is to some extent affected by the personality traits of the perceivers but that this relation depends on gender. In fact, the overall pattern of results shows that almost all of the significant, although low, correlations between perceived trustworthiness and personality traits turned to be significant only in the women’s subsample. To the best of our knowledge, no previous study analyzed the influence of gender in mediating the relationships between personality traits and trustworthiness judgments and future research should systematically investigate such relationships in order to gain a better understanding of the present findings.

Finally, gender seems to also affect the confidence in trustworthiness judgment. Specifically, after taking into account the gender differences in personality traits, the present results indicate that women were generally less confident than men in judging the trustworthiness of unfamiliar faces. In addition, women are more confident in judging trustworthiness of female faces compared to male faces. Among personality traits, as expected, Faith in Intuition is the trait that is most strongly related to confidence judgments: participants with higher faith in intuition tend to be more confident in their trustworthiness judgments. In contrast, more anxious individuals, especially women, are less confident in their judgments.

The present research is not exempt from limitations. First, the correlational nature of the study precludes us from concluding that agreeableness and aggression directly affect judgments of trustworthiness, and no causal inference can be made. A further limitation pertains to the fact that we investigated the effect of individual differences only on one dimension of facial appearance (i.e. trustworthiness). Future studies including the judgment of dominance may contribute to better understanding of the role of facial features and individual differences in first impression.

Despite these limitations, the present findings suggest that the effects of gender and personality traits of the observer are small in magnitude compared to the influence of facial appearance. However, consistent with a previous study [13], individual differences become more relevant depending on the intrinsic properties of the evaluated faces (e.g. the level of intrinsic trustworthiness of the face). Whom we decide to trust is a function not only of their facial features but also of our individual differences.
